# Predatory?

**DOI:** 10.1590/0102-311XEN024325

**Published:** 2025-03-24

**Authors:** Marilia Sá Carvalho, Luciana Dias de Lima, Luciana Correia Alves

**Affiliations:** 1 Programa de Computação Científica, Fundação Oswaldo Cruz, Rio de Janeiro, Brasil.; 2 Escola Nacional de Saúde Pública Sergio Arouca, Fundação Oswaldo Cruz, Rio de Janeiro, Brasil.; 3 Instituto de Filosofia e Ciências Humanas, Universidade Estadual de Campinas, Campinas, Brasil.

What is a predatory journal? Why is the discussion about such publications so relevant today? Why are these journals a cause for so much concern among academic institutions and scientists? What is the real impact of this problem? There is no clear definition of what constitutes a predatory journal ^1^. However, the Committee on Publication Ethics (COPE) ^2^ guidance on predatory publishing defines the practice as “*the systematic for-profit publication of purportedly scholarly content... in a deceptive or fraudulent way and without any regard for quality assurance*”.

The quality of articles published in *Science* or *Nature* is not questioned, despite retractions suggesting that quality is not always adequate ^3^. Likewise, despite being business-driven, with profit as the primary motivation, these journals maintain their credibility. In fact, the scientific publishing industry is one of the most profitable in the world - more profitable than Amazon, for example ^4^. What characterizes predatory publications?

One of the most evident aspects is the presence of false information about the journal, such as a website featuring a fake list of editors or prestigious researchers and forged impact indices. Another common practice is the publication of paid articles without a consistent, transparent review process, often including a fictitious peer review. Adding to this is aggressive marketing, primarily focused on the speed of publication, which appeals to researchers pressured by an evaluation system based on the “publish or perish” model, which directly impacts their chances of securing jobs or research funding. All of us, as scientists, regularly receive invitations to publish in journals we have never heard of. This rapid publication process aligns with researchers’ frequent complaints about the slow pace of article appraisal. However, it is worth considering: if peer review is the best guarantee of manuscript quality, and if the difficulty of obtaining good reviews is a well-known challenge for many journals, then such speed is, at the very least, questionable ^5^ ^,^ ^6^ ^,^ ^7^. Another striking issue is the high frequency of articles published by editors in the very journals they oversee. This was the case with the retracted article on the use of chloroquine for COVID-19 treatment, published in a journal in which one of the editors was also an author of the infamous paper.

Since the creation of the well-known list of predatory journals compiled by Jeffrey Beall - the American librarian who coined the term “predatory journals” - the impact of such journals in the scientific publishing world has grown significantly. The history of the Multidisciplinary Digital Publishing Institute (MDPI) is a prime example. In 2020, it was the 5th largest scientific publishing company in the world in terms of the number of articles published. In 2018, 10 senior editors of a journal within the group resigned due to pressure to accept manuscripts of questionable quality ^8^. By 2022, MDPI’s average acceptance rate was 50% of all submitted articles; thus almost all works were published, and quickly, ensuring maximum financial return. The average time between submission and acceptance is 35 days, regardless of the journal’s field. The reviews that are supposedly meant to ensure article quality are often produced by “review mills” ^9^. This system has been sustained by increasing the number of thematic issues, around 100 per journal, managed by guest editors. Whether they are called *Special Issues* (MDPI & Hindawi), *Research Topics* (Frontiers), or *Collections* (Springer Nature), this model is becoming increasingly common. Email invitations are no longer just for submitting articles but also for guest-editing special issues.

The boundary between predatory and legitimate journals is not well defined. The model based on special issues and guest editors is spreading rapidly. Elsevier, for instance, holds a webpage encouraging participation as a guest editor ^10^. The Frontiers group published 22% more articles in 2022 and 2023, and 134 new journals were launched from 2020 to 2023. In 2019 alone, 2,000 *Research Topics* were introduced ^11^. With 70% of articles published under this model in 2022, it has become the predominant publishing strategy for the group. As of 2024, *Frontiers in Medicine* presented 5,094 associated and guest editors ^12^. Publishers that initially had promising editorial proposals have changed over time. In 2015, 31 editors were removed from *Frontiers in Medicine* and *Frontiers in Cardiovascular Medicine* ^13^. An issue raised by the journals’ editors-in-chief was the autonomy of associate editors, approximately 150 academics, who operated without the involvement of the editors-in-chief or section editors ^13^. Despite this, the journal published high-quality articles.

How can researchers assess and choose where to publish their work? Over the last 20 years, the total number of published articles has tripled ^14^, and the number of journals indexed in Scopus has grown from 14,000 to 25,000 ^15^. There is no data available on non-indexed journals. A small portion of these are new journals dedicated to specific topics, often published in Global South countries. However, this is also where most predatory journals are found. The absence of a journal from major bibliographic indexes - especially when it has a high publication volume - is a factor worth considering ^16^. Authors can use checklists to avoid predatory journals, but some of these criteria may unintentionally exclude many journals outside the Global North, particularly those focused on Social Sciences and Humanities. A group of institutions focused on scientific publishing, including COPE and the Directory of Open Access Journals (DOAJ), created an initiative to support researchers in choosing reliable journals. Among the suggested criteria, perhaps the most insightful question is: Have you or a colleague found a recently published article in this journal to be relevant ^17^?

We cannot leave aside the core of this issue: the industry itself. This business sector is obviously attractive to all kinds of dubious dealings and unscrupulous operators. Even if a journal is eventually deemed predatory and loses credibility, the amount of financial resources already transferred is immeasurable. The attempt to define predatory journals with the statement, “*Predatory journals and publishers are entities that prioritize self-interest at the expense of scholarship...*” ^1^ (p. 211) applies just as well to all major academic publishers. At its core, the real issue is the appropriation of scientists’ work by commercial publishers ^18^. Addressing this problem is challenging. As long as research evaluation systems, whether by funding agencies or graduate programs, prioritize publishing increasing number of articles, predatory journals will continue to coexist. Some initiatives must advance in the short term. Around 700 research institutes, funding bodies, and international professional associations have formed the Coalition for Advancing Research Assessment (CoARA; https://coara.eu/) to propose reforms in research evaluation methods and processes. In Brazil, the Coordination for the Improvement of Higher Education Personnel (CAPES, acronym in Portuguese) has been working to refine the assessment of graduate programs by emphasizing the quality of research output. Maintaining scientific publishing under the control of institutions, associations, and research funders under diamond open access - in which neither authors nor readers pay - is the best strategy to move beyond the commercial and profit-driven publishing model. *Cadernos de Saúde Pública* takes pride in its diamond open access model, supported by Sergio Arouca National School of Public Health, Oswaldo Cruz Foundation.
