# Supplementary material for: Predatory?
Source: Cad Saude Publica. 2025 Mar 24;41(3):e00024325. doi: 10.1590/0102-311XEN024325 (PMC11940854; doi:10.1590/0102-311XEN024325)
Supplement: Translation

# Translation: Predatórias?

**DOI:** 10.1590/0102-311XPT024325

**Authors:** Marilia Sá Carvalho, Luciana Dias de Lima, Luciana Correia Alves

**Affiliations:** 1 Programa de Computação Científica, Fundação Oswaldo Cruz, Rio de Janeiro, Brasil.; 2 Escola Nacional de Saúde Pública Sergio Arouca, Fundação Oswaldo Cruz, Rio de Janeiro, Brasil.; 3 Instituto de Filosofia e Ciências Humanas, Universidade Estadual de Campinas, Campinas, Brasil.

O que é uma revista predatória? Por que a discussão sobre tais publicações está na ordem do dia? Por que são motivo de tanta preocupação entre instituições acadêmicas e cientistas? Qual é o impacto real do problema? Não existe uma definição clara do que é uma revista predatória ^1^. Mas a orientação do Comitê de Ética em Publicação (COPE, acrônimo em inglês) ^2^ define a prática como “*a publicação sistemática com fins lucrativos de conteúdo supostamente acadêmico... de forma enganosa ou fraudulenta e sem nenhuma consideração pela garantia de qualidade*”.

Ninguém questiona a qualidade dos artigos publicados na *Science* ou na *Nature*, mesmo que retratações indiquem que nem sempre a qualidade é adequada ^3^, mesmo que o modelo seja empresarial, tendo o lucro como principal motor. Aliás, a indústria da publicação científica é uma das mais lucrativas do mundo, mais do que a Amazon, por exemplo ^4^. Mas o que caracterizaria as publicações predatórias?

Um dos aspectos mais evidentes é a presença de informações mentirosas sobre a revista, uma página *web* com uma lista falsa de editores ou pesquisadores de prestígio e com índices de impacto inventados. Uma das suas práticas mais comuns é a publicação de artigos pagos, sem uma avaliação consistente, transparente e uma fictícia avaliação por pares. Junte-se a isso um marketing agressivo, baseado principalmente na rapidez da publicação, atraindo pesquisadores pressionados por um modelo de avaliação de suas carreiras baseada em *publish or perish* (publique ou pereça), que afeta diretamente a chance de ser contratado ou obter financiamento de projetos. Todos nós, cientistas, recebemos cotidianamente convites para publicar, rapidamente, em revistas sobre as quais nunca ouvimos falar. Essa rapidez vai ao encontro da frequente queixa de pesquisadores sobre a lentidão no processo de avaliação de artigos. Mas vale refletir: sendo a revisão por pares a melhor garantia da qualidade dos manuscritos publicados, e a dificuldade de obtenção de bons pareceres uma constatação comum a dezenas de revistas, a rapidez é no mínimo questionável ^5^ ^,^ ^6^ ^,^ ^7^. Chama a atenção também a frequência de artigos publicados por editores naqueles periódicos que comandam. Foi o caso do artigo retratado sobre o uso de cloroquina no tratamento da COVID-19, publicado em revista que tinha como editor um dos autores do infame artigo.

Desde a criação da famosa lista de revistas organizada por Jeffrey Beall, bibliotecário americano que cunhou o termo “predatório”, o peso dessas revistas no mundo da publicação científica aumentou de forma significativa. O histórico do *publisher* Instituto Multidisciplinar de Publicação Digital (MDPI, acrônimo em inglês), por exemplo, é paradigmático. Em 2020 era a quinta maior empresa de publicação científica do mundo quanto ao número de artigos publicados. Em 2018, dez editores seniores de um periódico do grupo renunciaram devido à pressão para aceitar manuscritos de qualidade duvidosa ^8^. A taxa de aceitação média do MDPI era, em 2022, 50% do total de artigos submetidos: publica-se praticamente tudo, rapidamente, garantindo o retorno financeiro máximo. O tempo entre submissão e aceitação é, em média, 35 dias, seja qual for a área temática da revista. Os pareceres que supostamente garantiriam a qualidade dos artigos são produzidos por “fábricas de pareceres” (*review mills*) ^9^. Essa dinâmica foi obtida por meio do aumento dos fascículos temáticos, cerca de 100 por revista, volume este sustentado por editores convidados. Sejam eles chamados *Special Issues* [Fascículos Especiais] (MDPI & Hindawi), *Research Topics* [Tópicos de Pesquisa] (Frontiers) ou *Collections* [Coleções] (Springer Nature), esse modelo é cada vez mais frequente. Os convites por e-mail não são mais para publicar um artigo, mas para editorar um número especial. 

A fronteira entre revista predatória e honesta não é tão bem definida. O modelo baseado em fascículos especiais e editores convidados está cada vez mais disseminado. Elsevier tem uma página na internet para estimular a participação de editores convidados ^10^. O grupo Frontiers publicou 22% mais artigos entre 2022 e 2023 e 134 novas revistas foram criadas entre 2020 e 2023. Em 2019 foram lançadas 2 mil publicações como *Research Topics* ^11^. Com 70% dos artigos publicados nesse modelo em 2022, pode-se afirmar que é esse o procedimento predominante do grupo. Somente na *Frontiers in Medicine*, em 2024, eram 5.094 editores associados e convidados ^12^. *Publishers* que começaram com propostas interessantes mudaram ao longo do tempo. Em 2015 foram afastados 31 editores da *Frontiers in Medicine* e *Frontiers in Cardiovascular Medicine* ^13^. Um dos aspectos questionados pelos editores-chefes das revistas foi a autonomia dos editores associados, aproximadamente 150 acadêmicos, sem envolvimento dos editores-chefes e editores de seção ^13^. Ainda assim, a revista publicou bons artigos.

Como o pesquisador pode avaliar e escolher onde publicar seu artigo? Nos últimos 20 anos o total de artigos publicados triplicou ^14^ e o número de revistas indexadas no Scopus passou de 14 mil para 25 mil ^15^. Não há dados sobre as revistas não indexadas. Uma pequena parte dessas são revistas novas, dedicadas a temas específicos, publicadas em países do Sul global. Mas é também onde encontraremos a maior parte das predatórias. A ausência nos principais indexadores bibliográficos, principalmente quando são revistas com grande volume de publicações, é um ponto a ser considerado ^16^. Autores podem utilizar checklists para evitá-las, mas alguns desses critérios excluiriam diversas revistas publicadas fora do eixo Norte global, principalmente as voltadas para as Ciências Sociais e Humanas. Um conjunto de instituições voltadas para a publicação científica, entre as quais o COPE e o Diretório de Periódicos de Acesso Aberto (DOAJ, acrônimo em inglês), criaram uma iniciativa para apoiar pesquisadores na escolha de revistas confiáveis. Entre diversas sugestões práticas disponibilizadas, talvez a pergunta mais interessante seja: você ou algum colega considerou relevante um artigo publicado recentemente nessa revista ^17^?

Não podemos deixar de lado a essência do problema: a indústria em si. Esse ramo de negócio é obviamente atrativo para todo o tipo de negociata e negociante sem escrúpulos. Ainda que em alguns anos a revista seja considerada predatória e perca espaço, o volume de recursos já transferido é incalculável. A tentativa de identificação de revistas predatórias resumida em “*Periódicos e editoras predatórias são entidades que priorizam interesses próprios em detrimento da pesquisa acadêmica...*” ^1^ (p. 211) serve perfeitamente para todos os *big publishers*. No fundo, a questão é mesmo essa: a apropriação do trabalho de cientistas por editoras comerciais ^18^. Não é uma tarefa fácil combater esse problema. Enquanto o modelo de avaliação dos pesquisadores pelas agências de fomento e dos programas de pós-graduação for publicar quantidades cada vez maiores de artigos, as revistas predatórias coexistirão. Algumas iniciativas precisam avançar no curto prazo. Cerca de 700 institutos de pesquisa, órgãos de financiamento, associações profissionais internacionais criaram uma associação denominada Coalizão para o Avanço da Avaliação de Pesquisa (CoARA, acrônimo em inglês; https://coara.eu/) para propor reformas de métodos e processos de avaliação de pesquisa. No Brasil, a Coordenação de Aperfeiçoamento de Pessoal de Nível Superior (CAPES) tem buscado aperfeiçoar a avaliação dos programas de pós-graduação valorizando a qualidade da produção. A manutenção da publicação científica por instituições, associações, financiadores de pesquisa, em um acesso aberto diamante, no qual nem autores nem leitores pagam, é a melhor estratégia para superação do modelo comercial e lucrativo da publicação científica. A publicação *Cadernos de Saúde Pública* se orgulha de ter acesso diamante, sustentado pela Escola Nacional de Saúde Pública Sergio Arouca, Fundação Oswaldo Cruz (ENSP/Fiocruz).

# Translation: ¿Depredadoras?

**DOI:** 10.1590/0102-311XES024325

**Authors:** Marilia Sá Carvalho, Luciana Dias de Lima, Luciana Correia Alves

**Affiliations:** 1 Programa de Computação Científica, Fundação Oswaldo Cruz, Rio de Janeiro, Brasil.; 2 Escola Nacional de Saúde Pública Sergio Arouca, Fundação Oswaldo Cruz, Rio de Janeiro, Brasil.; 3 Instituto de Filosofia e Ciências Humanas, Universidade Estadual de Campinas, Campinas, Brasil.

¿Qué es una revista depredadora? ¿Por qué está en discusión este tipo de publicaciones? ¿Por qué son tan preocupantes entre las instituciones académicas y los científicos? ¿Cuál es el impacto real del problema? No existe una definición exacta de qué es una revista depredadora ^1^. Sin embargo, Comité de Ética de Publicaciones (COPE, por su sigla en inglés) ^2^ dispone una guía en la cual define la publicación depredadora como “*una publicación sistemática con ánimo de lucro de contenidos supuestamente académicos... de forma engañosa o fraudulenta y sin ninguna garantía de calidad*”.

Nadie cuestiona la calidad de los artículos publicados en *Science* o *Nature*, aunque las retractaciones indican que la calidad no siempre es adecuada ^3^, o que el modelo es comercial en pro de beneficios. De hecho, la industria editorial científica es una de las más rentables del mundo, más rentable incluso que Amazon por ejemplo ^4^. Entonces, ¿qué caracterizaría a las publicaciones depredadoras?

Uno de los aspectos más evidentes es la presencia de información falsa sobre la revista, de una página web con una lista falsa de editores o investigadores de prestigio y de índices de impacto inventados. Entre sus prácticas más habituales destaca la publicación de artículos pagados, sin una revisión coherente y transparente, y una revisión por pares ficticia. A esto se suma un *marketing* agresivo centrado principalmente en la velocidad de la publicación, que atrae a investigadores presionados por un modelo de evaluación de sus carreras basado en *publish or perish* (publicar o perecer), lo que afecta directamente la posibilidad de ser contratado u obtener financiación para un proyecto. Todos los científicos recibimos invitaciones diarias para publicaciones rápidas en revistas que no conocimos. Esta velocidad coincide con la frecuente queja de los investigadores sobre la lentitud en el proceso de evaluación de los artículos. Sin embargo, cabe reflexionar que si la revisión por pares es la mejor garantía de calidad de los manuscritos publicados, y la dificultad de obtener dictámenes positivos es habitual en docenas de revistas, la velocidad es al menos cuestionable ^5^ ^,^ ^6^ ^,^ ^7^. También es notable la frecuencia de los artículos publicados por los editores en esas revistas. Este fue el caso del artículo sobre el uso de cloroquina en el tratamiento de la COVID-19, publicado en una revista cuyo editor es uno de los autores del infame artículo.

Desde la creación de la famosa lista de revistas organizada por Jeffrey Beall, el bibliotecario estadounidense que acuñó el término “depredador”, el peso de estas revistas en el ámbito de la publicación científica ha aumentado significativamente. La historia de la editorial Instituto Multidisciplinario de Edición Digital (MDPI, por su sigla en inglés) es paradigmática. En 2020 era la quinta empresa de publicaciones científicas más grande del mundo en relación con el número de artículos publicados. En 2018, 10 editores séniores de una revista del grupo renunciaron debido a la presión para aceptar manuscritos de dudosa calidad ^8^. La tasa promedio de aceptación de MDPI en 2022 fue del 50% del número total de artículos recibidos: prácticamente se publica todo de forma rápida, garantizando el máximo rendimiento financiero. El tiempo entre el envío y la aceptación lleva en promedio 35 días, independientemente del área temática de la revista. Los dictámenes que supuestamente garantizan la calidad de los artículos son producidos por “fábricas de dictámenes” (*review mills*) ^9^. Esta dinámica se obtuvo con el aumento de números temáticos, cerca de 100 por revista, un volumen que contó con el apoyo de editores invitados. Ya sean temáticas como *Special Issues* [Ediciones Especiales] (MDPI & Hindawi), *Research Topics* [Temas de Investigación] (Frontiers) o *Collections* [Colecciones] (Springer Nature), este es un modelo cada vez más frecuente. Las invitaciones por correo electrónico ya no son para publicar un artículo, sino para editar un número especial. 

El límite entre una revista depredadora y una revista honesta no está tan bien definido. El modelo basado en números especiales y editores invitados está cada vez más extendido. Elsevier tiene una página web para fomentar la participación de los editores invitados ^10^. El grupo Frontiers publicó un 22% más de artículos entre 2022 y 2023; y se crearon 134 nuevas revistas entre 2020 y 2023. En 2019, se lanzaron 2.000 publicaciones como *Research Topics* ^11^. Con el 70% de los artículos publicados bajo este modelo en 2022, se constata que este es el procedimiento predominante del grupo. En 2024, *Frontiers in Medicine* contaba con 5.094 editores asociados e invitados ^12^. Las editoriales que empezaron con propuestas interesantes han cambiado con el tiempo. En 2015 se dimitieron 31 editores de *Frontiers in Medicine* y *Frontiers in Cardiovascular Medicine* ^13^. Uno de los aspectos cuestionados por los editores en jefe de las revistas fue la autonomía de los editores asociados, cerca de 150 académicos, sin la participación de los editores en jefe y de los editores de sección ^13^. Sin embargo, la revista publicó buenos artículos.

¿Cómo el investigador puede evaluar y elegir dónde publicar su artículo? En los últimos 20 años, el número total de artículos publicados se ha triplicado ^14^ y el número de revistas indexadas en Scopus ha aumentado de 14.000 a 25.000 ^15^. No hay datos sobre revistas no indexadas. Una parte de estas son nuevas revistas dedicadas a temas específicos publicados en países del Sur global. En esta región encontraremos la mayoría de las revistas depredadoras. La ausencia en los principales indexadores bibliográficos, especialmente cuando se trata de revistas con un gran volumen de publicaciones, es un punto considerable ^16^. Los autores pueden utilizar listas de verificación para evitar estas revistas, pero algunos de estos criterios excluirían varias revistas publicadas fuera del eje Norte global, especialmente aquellas centradas en las Ciencias Sociales y las Humanidades. Un grupo de instituciones enfocadas en la publicación científica, entre ellas COPE y el Directorio de Revistas de Acceso Abierto (DOAJ, por su sigla en inglés), crearon una iniciativa para apoyar a los investigadores en la elección de revistas confiables. Entre el conjunto de criterios sugeridos, quizás la pregunta más interesante sea: ¿Usted o algún colega han considerado un artículo relevante publicado recientemente en esta revista ^17^?

No podemos dejar de lado la esencia del problema: la propia industria. Evidentemente, esta línea de negocio es atractiva para todo tipo de negociantes sin escrúpulos. Aunque con el tiempo la revista pasará a ser considerada depredadora y perderá espacio, el volumen de recursos ya transferidos habrá sido incalculable. El intento de identificar las revistas depredadoras resumido em “*Las revistas y editoriales depredadores son entidades que priorizan sus propios intereses en detrimento de la investigación académica*...” ^1^ (p. 211) se adapta perfectamente a todas las grandes editoriales. De hecho, el problema es este: la apropiación del trabajo de los científicos por parte de las editoriales comerciales ^18^. No es una tarea sencilla combatir este problema. Mientras el modelo de evaluación de los investigadores por parte de las agencias de financiación y de los programas de posgrado tenga como objetivo publicar cada vez más artículos, las revistas depredadoras coexistirán. Algunas iniciativas necesitan avanzar en el corto plazo. Cerca de 700 institutos de investigación, agencias de financiamiento y asociaciones profesionales internacionales han creado una asociación llamada Coalición para el Avance de la Evaluación de la Investigación (CoARA, por su sigla en inglés; https://coara.eu/) con el objetivo de proponer reformas en los métodos y procesos de evaluación de la investigación. En Brasil, la Coordinación para el Mejoramiento del Personal de Educación Superior (CAPES, por su sigla en portugués) viene buscando mejorar la evaluación de los programas de posgrado al valorar la calidad de la producción. El mantenimiento de la publicación científica por parte de instituciones, de asociaciones, de agencias de financiamiento de investigación en un acceso abierto diamante, en el que ni los autores ni los lectores pagan, es la mejor estrategia para superar el modelo comercial y rentable de la publicación científica. *Cadernos de Saúde Pública* se enorgullece de tener acceso diamante con el apoyo de la Escuela Nacional de Salud Pública Sergio Arouca, Fundación Oswaldo Cruz.
